# Supply and Demand in mHealth Apps for Persons With Multiple Sclerosis: Systematic Search in App Stores and Scoping Literature Review

**DOI:** 10.2196/10512

**Published:** 2018-05-23

**Authors:** Guido Giunti, Estefanía Guisado Fernández, Enrique Dorronzoro Zubiete, Octavio Rivera Romero

**Affiliations:** ^1^ Salumedia Tecnologias Seville Spain; ^2^ University of Oulu Oulu Finland; ^3^ University College Dublin Dublin Ireland; ^4^ Universidad de Sevilla Seville Spain

**Keywords:** multiple sclerosis, mHealth, fatigue, fatigue management, apps, gamification, user-centered design, usability, physical activity, eHealth, chronic conditions

## Abstract

**Background:**

Multiple sclerosis (MS) is a non-curable chronic inflammatory disease of the central nervous system that affects more than 2 million people worldwide. MS-related symptoms impact negatively on the quality of life of persons with MS, who need to be active in the management of their health. mHealth apps could support these patient groups by offering useful tools, providing reliable information, and monitoring symptoms. A previous study from this group identified needs, barriers, and facilitators for the use of mHealth solutions among persons with MS. It is unknown how commercially available health apps meet these needs.

**Objective:**

The main objective of this review was to assess how the features present in MS apps meet the reported needs of persons with MS.

**Methods:**

We followed a combination of scoping review methodology and systematic assessment of features and content of mHealth apps. A search strategy was defined for the two most popular app stores (Google Play and Apple App Store) to identify relevant apps. Reviewers independently conducted a screening process to filter apps according to the selection criteria. Interrater reliability was assessed through the Fleiss-Cohen coefficient (k=.885). Data from the included MS apps were extracted and explored according to classification criteria.

**Results:**

An initial total of 581 potentially relevant apps was found. After removing duplicates and applying inclusion and exclusion criteria, 30 unique apps were included in the study. A similar number of apps was found in both stores. The majority of the apps dealt with disease management and disease and treatment information. Most apps were developed by small and medium-sized enterprises, followed by pharmaceutical companies. Patient education and personal data management were among the most frequently included features in these apps. Energy management and remote monitoring were often not present in MS apps. Very few contained gamification elements.

**Conclusions:**

Currently available MS apps fail to meet the needs and demands of persons with MS. There is a need for health professionals, researchers, and industry partners to collaborate in the design of mHealth solutions for persons with MS to increase adoption and engagement.

## Introduction

Multiple sclerosis (MS) is a noncurable chronic inflammatory condition of the central nervous system that affects more than 2 million people around the world [1]. Both Europe and North America are considered high prevalence regions for MS [2]. MS impacts mental and physical aspects, the most common symptoms being overwhelming fatigue, altered sensation, cognitive problems, visual disturbances, spasticity, pain, and bladder problems [3]. Persons with MS are negatively affected in their quality of life [4], with periods during which these symptoms worsen [3,5]. They generally have a similar life expectancy as the general population and have to learn to manage their symptoms over long periods of time. It is crucial then for persons with MS to be active patients, more engaged with their health [3]. Living with MS often requires individuals to be more engaged with their health as their quality of life is affected in many ways [6] leading to self-management needs [3]. Current research shows that in order to successfully manage chronic conditions, patients require support both to learn about and manage their symptoms and problems [7-9].

Currently, “the delivery of health care or health related services through the use of portable devices,” or mHealth [10], is increasingly being used in many chronic diseases such as diabetes [11], cancer [12], and hypertension [13]. Studies have explored how different health care stakeholders such as patients and their social group, health care professionals, and caregivers can benefit from the use of those technologies [14]. Mobile devices are ubiquitous, being less invasive in day-to-day situations, allowing the tracking of persons’ activities, providing real-time feedback, and with a high cost-effectiveness [15-17]. Together with the number of mobile devices per capita, the use of mobile software apps for health and well-being promotion has increased in recent years [18]. Many of them are focused on supporting persons with chronic diseases in managing their conditions. In order to be effective, however, mHealth solutions need to meet users’ needs and preferences to provide appropriate features and contents and ensure higher adoption and adherence rates [19-21]. In the case of MS and because of the variety of symptoms and problems that persons with MS may suffer, mHealth solutions should also take into consideration the particular and specific needs that they have. Additionally, the use of game elements in non-game contexts such as health apps (ie, gamification [22]) is now openly used as a strategy for increasing user engagement [23-26]. The current gamification prevalence in MS apps is unknown.

In previous stages of our work, we conducted a qualitative study to identify the desired features and characteristics for mHealth solutions for persons with MS [27] and performed a preliminary review of MS mHealth apps [28]. In this paper, we revise and expand on that work by conducting a methodological review of the commercially available mHealth solutions for persons with MS in the most popular app stores, to assess whether those apps are meeting their needs and preferences. This study addresses the following research questions (RQ):

RQ1: What health apps are available for persons with MS?

RQ2: What is the intended purpose of these health apps?

RQ3: What stakeholders are behind these health apps?

RQ4: What features do these health apps offer?

RQ5: How prevalent is gamification in these health apps?

## Methods

### Study Design

The methodology used in this study is based on two approaches: scoping review and systematic review methodologies. Scoping review methodology aims to map the key concepts underpinning a research area especially where an area has not been reviewed comprehensively before [29-31]. Systematic review methodology has a clearly formulated question that uses systematic and explicit methods to identify, select, and critically appraise relevant research, and to collect and analyze data [32]. These approaches have been used in the past to assess features and content of mHealth apps [33-36].

A search strategy was defined to identify all potentially relevant health apps. Since the objective of this study is to identify all apps that target persons with MS, we defined “Multiple Sclerosis” as the main search term. In September 2017, two reviewers (OR-R and ED-Z) used these keywords to look for matching apps whether in titles or descriptions. The two most popular app stores were searched: Google Play Store and Apple App Store. These stores were explored in their versions in the United States and in Spain. Searches for Google Play Store were conducted through its website, taking steps to ensure that no previous searches or cookies influenced our results. The Apple App Store was searched using iTunes App installed on two iOS devices (iPad and iPhone), one for each locale (US and Spain).

### Selection Criteria

Apps were included if the title or store description of the app contained specific mention of MS. Duplicate entries were removed and 2 reviewers (OR-R and ED-Z) evaluated the eligibility of the found apps to include only those that met the inclusion criteria and had none of the exclusion criteria. Health apps that had versions in different operating systems were considered the same app [37] and only the Android version was included for analysis. Disagreements were resolved by consensus involving a third reviewer when necessary. The Fleiss-Cohen interrater coefficient was calculated showing high reliability (*k*=.885).

#### Inclusion Criteria

We defined the following inclusion criteria: (1) the title or description referred to MS, and (2) it was present in the versions of Google Play Store and Apple App Store for the US or Spain.

#### Exclusion Criteria

Apps resulting in the searches were excluded if they met at least one of the following conditions: the title or description was not written in English or Spanish, user interface was not available in English or Spanish, the app was intended for other health conditions, or they were duplicates from the same store.

**Desired features and characteristics for mHealth solutions for persons with multiple sclerosis (MS).**

**Customizable goal setting:** challenges need to be tailored to the specific person with MS characteristics

**Energy profiles and fatigue management: i**nformation and tools that help users in managing their day-to-day activities

**Patient education:** offer verified information that is helpful and reliable

**Data visualization:** information must be presented in a way that is meaningful to persons with MS

**Positive feedback system:** rewards and incentives for completing tasks and objectives

**Activity tracking:** register metrics such as distance walked or run, calorie consumption, heartbeat, and quality of sleep among others

**Exercise library:** a collection of different activities beneficial to persons with MS, like fitness or relaxation techniques that can be selected

**Game-like attitude:** playfulness is a mindset whereby people approach activities as something not serious, in a way that is highly pleasurable and motivating

**Strong evidence base:** features and information offered should have a solid scientific foundation

**Remote monitoring:** health care providers can follow persons with MS progress and give feedback

**Optional Sociability:** ability to opt out of social media features like messaging, feeds, or other kinds of social comparisons

**Reminders system:** notifications that remind persons with MS to engage in activities

**Personal data management:** access to personal information and data defined by the user case by case

### Data Extraction and Classification

Apps meeting the eligibility criteria were downloaded and installed into testing devices (Android: LG G4 and Motorola G5; iOS: iPad 2 and iPhone 5) for data extraction. GG, ED-Z, and OR-R independently manually extracted data from the included apps.

Descriptive characteristics were extracted where available: (1) app platform, last update date, price, ratings, number of ratings, number of downloads, languages, and developer agency; (2) intended purpose; (3) feature match with previous study; (4) and presence of game elements as defined by Johnson et al [38].

Developer agencies were coded into one category following our classification scheme published in [28] and similar to ones present in other studies [36]. The classification scheme is described below:

- Health care‒related agency: Hospitals, clinics, or governmental organizations directly related to health care (ie, public health branches)
- Pharmaceutical company: Entities with commercial purposes to research, develop, market, or distribute drugs in the context of health care
- Governmental agency: Any governmental agency or organization not directly involved in health care (ie, IT departments)
- Nongovernmental agency: Any organization that is neither part of a government nor a conventional for-profit business such as societies or organizations that specialize both in general health improvement as well as illness-specific objectives and offer support groups (ie, patient empowerment organizations)
- Educational organization: Any educational organization such as universities, colleges, libraries, or schools not directly related to health care (ie, science school projects)
- Conferences and journals: Scientific journals, patient, or medical conferences
- Small and medium-sized enterprises: Start-ups, software developing companies, or any other private organizations that identified themselves as an enterprise and not individuals (ie, digital health start-ups)
- Individuals: Developers or uploader entities who are listed as individuals or have not identified themselves as enterprises (ie, John Smith)

Classification for the app’s main purpose followed the scheme published in [28] and shown below:

- Awareness-raising: Tools to raise public recognition of MS as a problem, tools for fundraising, etc.
- Disease and treatment information: Provide general information about MS (eg, disease or treatment options)
- Disease management: Provide information and practical tools to deal with the medical, behavioral, or emotional aspects of MS
- Support: Provide access to peer or professional assistance

MS app features were matched with the desired features found in our previous study [27] shown in Textbox 1.

## Results

### Selection

**Figure 1:**
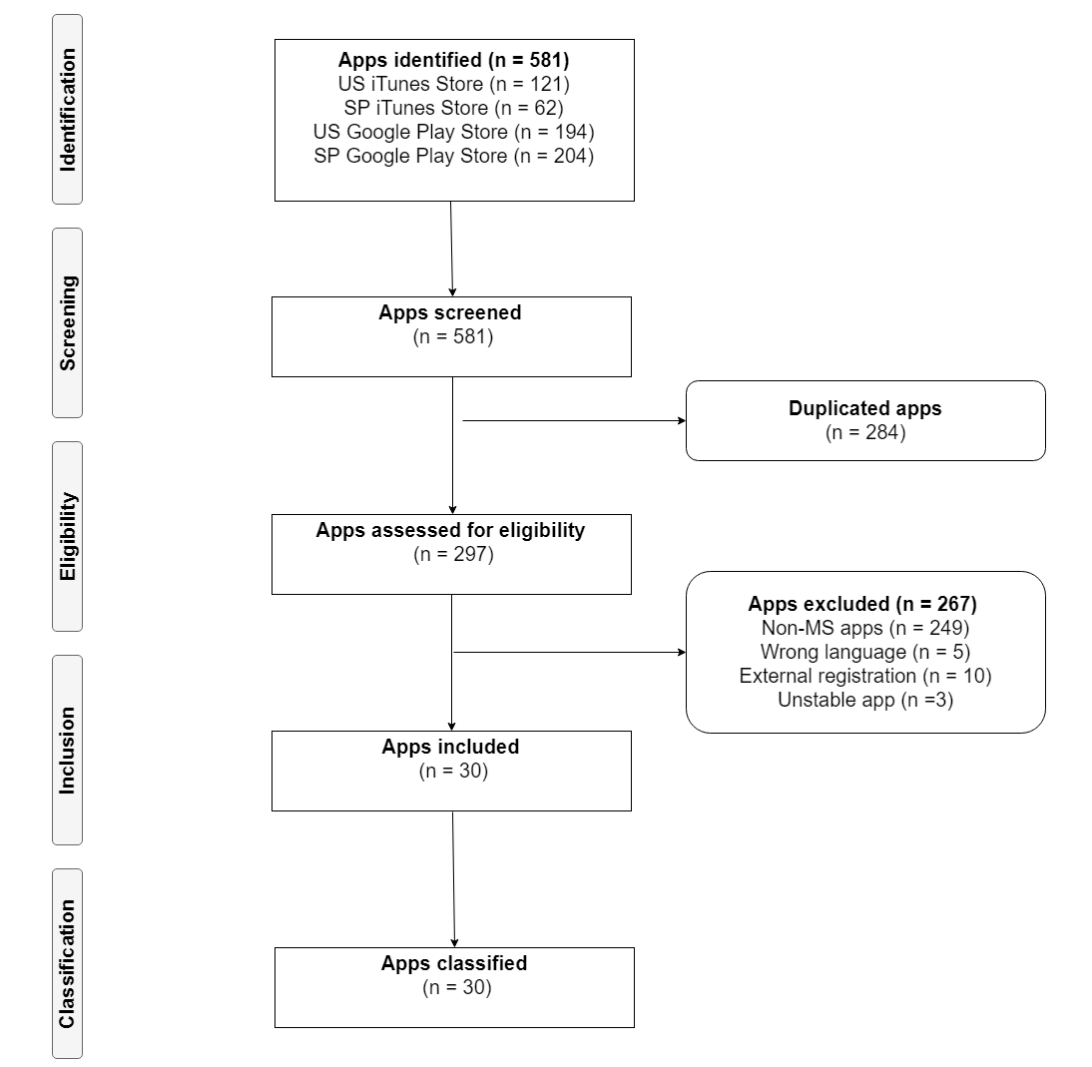
Study flow.

The searches in the Android and iOS markets yielded 581 potentially relevant apps. Removing duplicates and applying the selection criteria resulted in a total of 30 unique MS apps. As mentioned in the selection criteria section, only the Android versions of apps that were present in both platforms were included for analysis. However, due to technical problems with two of these multisystem apps, the iOS versions were included instead (19 Android apps and 11 iOS apps). Additionally, we found that some apps required registration outside of the app interface, so we attempted registration on these sites and excluded those that were private. Figure 1 shows the overall study flow including the number of apps explored in each stage.

### General Characteristics

The list of the included apps is shown in Table 1. A summary of the general characteristics of MS apps is shown in Table 2.

The large majority of apps were free to download (26/30, 87%) versus paid apps (4/30, 13%). The prices of paid apps ranged from US $0.99 to $4.99. Using the established 5-star rating system, most Android apps had good ratings with 3 or more stars (16/19, 84%). In relation to the number of downloads, at least half of them had over 500 downloads (58% of the included Android apps, 11/19). The most downloaded Android app, “Multiple Sclerosis Support,” had more than 10,000 downloads. On the other hand, the majority of iOS apps had no ratings (7/11, 64%), and no data on the number of downloads were available as Apple does not provide this information.

The majority of MS apps were available in English (27/30, 90%) with a small number of apps available only in Spanish (3/30, 10%).

### App Purpose and Affiliation

Based on our classification schemes, disease management apps were the most predominant (13/30, 43%), followed by disease and treatment information apps (11/30, 37%; see Table 3). Information about the apps affiliation is shown in Table 3.

**Table 1:** List of multiple sclerosis (MS) apps.

Name	Platform
Basic MS Explorer	Android / iOS
Becare MS Link	Android
Con la EM	Android
Control EM	Android
Cure MS	iOS
EM All in One	Android / iOS
Healthstories MS	Android / iOS
MCAMS	iOS
MS Buddy: Multiple Sclerosis	Android / iOS
MS journal	iOS
MS Mate	Android
MS Self Multiple Sclerosis App for MS Patients	Android / iOS
MS Topography	iOS
MSFocus Radio	Android / iOS
MSstation	iOS
Multiple Esclerosis	Android
Multiple Sclerosis	Android
Multiple Sclerosis	Android
Multiple Sclerosis 101-Treatment and Recovery Tips	iOS
Multiple Sclerosis Attack App	Android / iOS
Multiple Sclerosis Chat	Android
Multiple Sclerosis Messenger	Android
Multiple Sclerosis Support	Android
My MS Conversations	Android / iOS
My MS Manager	Android / iOS
My MS-UK	Android / iOS
My Sidekick	iOS
Pre-meet	iOS
Rebilink	iOS
Understanding MRI: Multiple Sclerosis	Android / iOS

### App Features

Apps were further analyzed to assess which features were present. Table 3 shows features included in the studied apps. “Patient education” was the most prevalent feature in the dataset, followed by “Social media” and “Data visualization.”

The majority of MS apps used mobile phone media capabilities (text, video, and audio) to deliver content to the user (17/30, 57%). Other features such as data visualization (7/30, 23%), social media (7/30, 23%), and reminders (6/30, 20%) were frequently present. Less popular features were personal data management (3/10, 7%), activity tracking (3/30, 10%), the presence of exercise libraries (2/30, 7%), remote monitoring (1/30, 3%), and energy and resource management (1/30, 3%).

#### Patient Education

Information for patient education was abundant but references to source materials was scarce (only present in one third of MS apps). Table 3 shows media format selection.

#### Social Media

The social media features included in the studied apps provided content sharing features through different social media networks. In 5 of the apps with social media features, socialization was optional, allowing users to decide whether to use it to share information with others. Some apps had their own social networks exclusively for patients, such as patient’s forums, chats, or specific platforms, while the rest offered standard social media outlets like Facebook and Twitter.

#### Data Visualization

Almost a quarter of the apps (7/30, 23%) featured some kind of user-generated data visualization. The data were usually obtained from in-app surveys and questionnaires.

#### Reminders

Only 6 of the apps had some sort of reminder system that allowed the user to set the notification frequency according to their preferences. The reminders helped users to remember to take medications (5/6), keep track of medical appointments (3/6), use activity tracking (1/6), and note down questions to ask health care professionals in upcoming visits (1/6). Other notifications such as content updates were also included in one of those apps.

#### Personal Data Management

Entering personal data information was among the first things asked by 10 of the apps. However, allowing users to decide or manage how their personal data was used was a somewhat infrequent feature. Only two apps allowed the user any choice regarding what data could be shared. None of the apps offered any option for the user to choose with whom data were shared. Only two apps included any kind of personalization of content or experience based on personal data collected.

#### Activity Tracking

Regarding activity tracking, only one MS app provided connectivity to external sensors (in this case Fitbit devices) while the rest relied on integrated capabilities within the mobile phone.

#### Exercise Library

Only two apps included a physical exercise library, sorting proposed exercises into categories such as body part and physical abilities, or showing lists of exercises without any classification or frame of reference.

#### Energy and Resource Management

**Figure 2:**
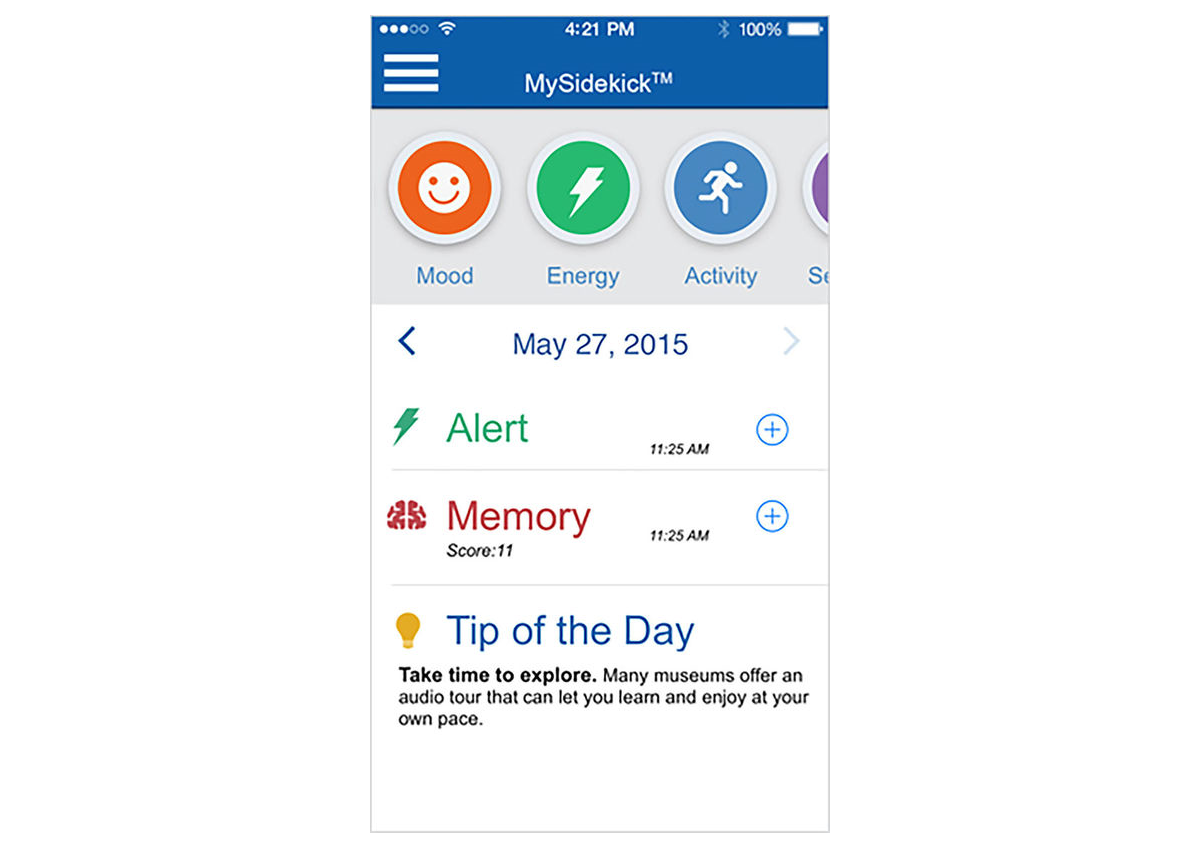
Example of energy and resource management multiple sclerosis app.

Only one app called “My sidekick” (Figure 2) dealt with energy and resource management for persons with MS in any capacity. It included a user profile for collecting information about mood, symptom-related sensations, energy level, and activities carried out for the day.

**Table 2:** General characteristics summary.

Characteristics		Android (n=19), n (%)		iOS (n=11), n (%)		Total (N=30), n (%)	
**Commercialization**							
	Free	18 (95)		8 (73)		26 (87)	
	Paid	1 (5)		3 (27)		4 (13)	
	Rated	18 (95)		4 (36)		22 (73)	
**Rating (number of stars)^a^**							
	—	1 (5)		7 (64)		8 (27)	
	⋆	0 (0)		0 (0)		0 (0)	
	⋆⋆	2 (10.5)		1 (9)		3 (10)	
	⋆⋆⋆	7 (37)		1 (9)		8 (27)	
	⋆⋆⋆⋆	5 (26)		1 (9)		6 (20)	
	⋆⋆⋆⋆⋆	4 (21)		1 (9)		5 (17)	
**Number of downloads^b^**							
	1-5	0 (0)		–		0 (0)	
	5-10	1 (5)		–		1 (3)	
	10-50	1 (5)		–		1 (3)	
	50-100	2 (10.5)		–		2 (7)	
	100-500	4 (21)		–		4 (13)	
	500-1000	2 (10.5)		–		2 (7)	
	1000-5000	6 (31.5)		–		6 (20)	
	5000-10,000	2 (10.5)		–		2 (7)	
	10,000-50,000	1 (5)		–		1 (3)	
	Not available	0 (0)		11 (100)		11 (37)	

^a^Apps are rated based on a 5-star rating system.

^b^Number of downloads are provided as a range by Google; this information is not provided for iOS apps.

**Table 3:** Characteristics of multiple sclerosis apps.

Characteristics		n (%)
**Purpose**		
	Disease management	13 (43.3)
	Disease and treatment information	11 (36.6)
	Support	5 (16.6)
	Awareness-raising	1 (3.3)
**Origins**		
	Small and medium enterprises	24 (80.0)
	Pharmaceutical companies	3 (10.0)
	Health care­–related agencies	1 (3.3)
	Nongovernmental agencies	1 (3.3)
	Individuals	1 (3.3)
**Most prevalent features**		
	Patient education	17 (36.2)
	Social media	7 (14.9)
	Data visualization	7 (14.9)
	Reminders	6 (12.7)
	Personal data management	3 (6.4)
	Activity tracking	3 (6.4)
	Exercise library	2 (4.3)
	Remote monitoring	1 (2.1)
	Energy and resource management	1 (2.1)
**Media formats**		
	Text	14 (46.7)
	Audio	7 (23.3)
	Video	2 (6.7)
**Game elements**		
	Progress representation	4 (13.3)
	Goal-setting	3 (10.0)
	Rewards system	3 (10.0)
	Social interaction	2 (6.6)
	Avatars	2 (6.6)
	Leaderboards	1 (3.3)
	Narrative	0 (0)

#### Remote Monitoring

“My MS Manager” app was the only app offering any kind of remote monitoring feature. This app presented users with the option to provide access on symptoms, laboratory results, medications, and side effects with the health care professionals who care for them.

### Gamification

The prevalence of different game elements is presented in the Table 3. In general terms, the MS apps included in this study did not make use of gamification. The most popular game design elements were progress representations (progress, feedback, and levels), goal setting (goals and challenges) and rewards, social interaction opportunities, and avatars. No apps included narrative devices as a gamification technique.

## Discussion

### Principal Findings

To our knowledge, this is the first study to provide an in-depth analysis of mHealth apps for persons with MS available to consumers and contrast it to their reported needs. As it stands, it captures the current landscape for the ecosystem and the active stakeholders involved in it. mHealth apps for MS were classified according to their main features and characteristics. This study also explored the information presented to users and assessed the presence of references to source material to understand its reliability. The current work is also the first to evaluate the extent of gamification elements present in MS mobile apps.

In summary, a total of 30 unique health apps were identified across the two most popular app stores (Google Play and Apple App Store). A similar number of apps were found in both stores. The majority of the apps dealt with disease management and disease and treatment information. Most apps were developed by small and medium-sized enterprises, followed by pharmaceutical companies. Patient education and personal data management were among the most frequently included features in these apps. On the other hand, energy and resource management, and remote monitoring were often not present. Very few MS apps used gamification elements.

### Comparison With Prior Work

Patient education is an essential strategy in the management of MS [3]. Self-management interventions typically focus on teaching skills, such as problem solving and decision making that are relevant to promoting engagement in single and multiple behaviors to manage single or multiple symptoms [39]. It is through proper patient education that persons with MS may achieve optimal outcomes and improvements in their quality of life [40]. In this review, patient education features were found to be among the most prevalent. The majority of the apps approached this topic providing information about MS and through some amount of disease management features.

Despite the fact that educational content was included in most of the analyzed apps, reliability of those solutions could use improvement. First, most MS apps did not reference the sources of their contents. Second, as shown in Table 3 most of the current mHealth apps for MS have been developed by small and medium-sized enterprises with little involvement from health care agencies or nongovernmental organizations. This could be an important factor preventing adoption as MS patients have expressed concerns about the entities responsible for health apps [27]. This was also present in our previous study [27], as persons with MS claimed that “professional endorsement” was a high priority factor for accepting online health information or mHealth solutions. While it is possible that health care professionals may have been involved in the development and design of these mHealth apps, such information is not disclosed or easily accessible. Reliability of content is a common problem in mHealth [28,36,37], as a large number of health apps for patients are not adequate: some do not have correct information, lack transparency, or are inconsistent regarding personal data usage and storage [41]. Further exploration about data security issues should be undertaken to understand how these apps deal with these issues.

Regarding the way the available information was shown in some apps, such as MSFocus radio or Basic MS explorer for example, there seemed to be issues on how content was presented to users. The information did not have a proper information architecture as topics were shown mixed in a timeline feed without any search feature available. This issue has been reported to reduce usability and may result in a poor user experience decreasing the adoption rate and users’ engagement [42]. The way that information is presented to users is key for them to be able to relate to it. Representing data visually is an important feature as it allows patients to relate in a meaningful way [43]. Of all the studied apps, only 4 offered some kind of visual reporting.

The chronic care model [44] emphasizes the role of patient as being their own caregiver and the importance of a collaborative partnership between patient and provider and the family and community support. Most of the studied apps do not provide family or other members of the social group a role or use case; the closest this feature got to that level was offering social media connectivity. Additionally, the current solutions do not offer a place for collaborative work with health care providers, which was also identified as a desirable feature for persons with MS [27].

Persons with MS experience severe levels of disability along their life and can suffer disabling fatigue. The lack of mHealth solutions that addresses fatigue management is intriguing and could take advantage of potentially interesting approaches that use mobile phones to monitor sleep cycles and promote physical activity [45,46]. The mobile phone’s embedded sensors and its capabilities for using external sensors such as step counters and other wearable devices also present an opportunity for further research [47,48]. Monitoring and influencing physical activity using mobile phones has been proven as a tool to provide average-to-excellent levels of accuracy for different behaviors and as a valid tool for assessment of physical activity [49]. However, only two of the apps made use of these capabilities while the rest relied on manually entered data or none at all.

Evidence suggests that physical activity helps people with MS stay active, reduces MS symptoms, and improves cognitive abilities but still many individuals with MS avoid physical activity [50-54]. Physical activity for MS patients is an important factor for improving and managing the physical demands of MS. Having a variety of exercise programs was a highlighted need that seems to be unmet. In our previous study [27], lack of enjoyment was a big de-motivator for physical activity for persons with MS: many mentioned that perhaps the use of game elements or having a game-like attitude to physical activity would make it more appealing. Only a few apps included gamification elements that could facilitate user retention through the motivation. Future mHealth designers could take some direction from the current gamification design guidelines available [55].

Personal data are often collected but seldom used to improve personalization or remote monitoring functionalities. Issues regarding data confidentiality have been raised [27], but none of the included apps allowed users to select with whom they could share their data. This is of particular note, considering that the second largest group of developers was pharmaceutical companies, and how in our previous study [27], both health care professionals and persons with MS were concerned about the involvement of these type of companies.

### Limitations

This study does have limitations. One limitation lies with the way search algorithms work, as they return partial matches as well as full matches, so some apps may have been missed in our search. Another limitation is that we relied on app store descriptions for identification. It is also possible that in some instances developers disclose sources, features, or affiliations once in-app; this seems unlikely given that such features are positive selling points and would be highlighted if present.

The focus of our study revolved around apps available in app stores for the United States and Spain, which might have excluded potentially relevant apps (published in the United Kingdom or Canadian stores, for example). The structural differences between stores also made it impossible to compare certain aspects (eg, iTunes does not disclose number of downloads per app). Restricting app stores to Apple and Android-based mobile phones could also introduce a selection bias as proportions might differ in less popular platforms such as Windows or Blackberry phones.

Additionally, further assessment could have been undertaken for each health app. App quality assessments such as the Mobile App Rating Scale (MARS) methodology [56] are available, and theoretical framework regarding the technology adoption such as the Technology Acceptance Model (TAM) model [57] or Unified Theory of Acceptance and Use of Technology (UTAUT) [58] were considered but found to be beyond the scope of this study.

Finally, while the presence of reference to source materials was explored, the validity of those sources was not assessed in relation to evidence-based guidelines. This is an interesting field for further exploration.

### Conclusions

This study analyzed current multiple sclerosis health apps available to consumers. The use of these mHealth apps is appealing, but the current landscape does not seem to match the needs of persons with MS. The lack of involvement from health care professionals and lack of sound quality information is still a major issue. This presents an interesting opportunity to improve these patient-facing apps and address the lack of health care providers’ end of the equation. Features such as social support, exercise library, and energy and resource management are not present in most of the MS apps. Among the few MS apps available, most were rated positively, indicating that there is perhaps a strong interest in mHealth solutions for MS. It would be interesting for future research to find more ways of personalizing MS apps to persons with MS specific needs.

## Abbreviations

mHealth: mobile health
MS: multiple sclerosis
RQ: research question
